# Comparison of Machine Learning Algorithms and Hybrid Computational Intelligence Algorithms for Rehabilitation Classification and Prognosis in Reverse Total Shoulder Arthroplasty

**DOI:** 10.3390/bioengineering12020150

**Published:** 2025-02-05

**Authors:** Sotiria Vrouva, George A. Koumantakis, Varvara Sopidou, Petros I. Tatsios, Christos Raptis, Adam Adamopoulos

**Affiliations:** 1 Physiotherapy Department, School of Health and Care Sciences, University of West Attica (UNIWA), 12243 Athens, Greece; gkoumantakis@uniwa.gr (G.A.K.); ptatsios@uniwa.gr (P.I.T.); 2 Department of Physical Therapy, 401 Army General Hospital of Athens, 11525 Athens, Greece; 3 Medical Physics Laboratory, Department of Medicine, Democritus University of Thrace, 68100 Alexandroupolis, Greece; craptis@med.duth.gr (C.R.); adam@med.duth.gr (A.A.); 4 Department of Biomedical Sciences, School of Health and Care Sciences, University of West Attica (UNIWA), 12243 Athens, Greece; vsopidou@uniwa.gr

**Keywords:** reverse total shoulder arthroplasty prognosis, genetic algorithms, hybrid machine learning algorithms

## Abstract

Despite the increasing application of machine learning and computational intelligence algorithms in medicine and physiotherapy, accurate classification and prognosis algorithms for postoperative patients in the rehabilitation phase are still lacking. The present study was carried out in two phases. In Phase I, classification performance of simple machine learning algorithms applied on data of patients suffering of reverse total shoulder arthroplasty (RTSA), examining algorithms’ classification accuracy and patients’ rehabilitation prognosis. In Phase II, hybrid computational intelligence algorithms were developed and applied in order to search for the minimum possible training set that achieves the maximum classification and prognostic performance. The data included features like age and gender, passive range of available motion of all movements (preoperative and postoperative), visual analog pain scale (preoperative and postoperative), and total rehabilitation time. In Phase I, K-nearest neighbors (ΚΝΝ) classification algorithm and K-means clustering algorithm (GAKmeans) were applied. Also, a genetic algorithm (GA)-based clustering algorithm (GAClust) was also applied. To achieve 100% performance on the test set, KNN used 80% of the data in the training set, whereas K-means and GAClust used 90% and 53.3%, respectively. In Phase II, additional computational intelligence algorithms were developed, namely, GAKNN (Genetic Algorithm K-nearest neighbors), GAKmeans, and GA2Clust (genetic algorithm-based clustering algorithm 2), for genetic algorithm optimization of the training set. Genetic algorithm optimization of the training set using hybrid algorithms in Phase II resulted in 100% performance on the test set by using only 35% of the available data for training. The proposed hybrid algorithms can reliably be used for patients’ rehabilitation prognosis.

## 1. Introduction

Reverse total shoulder arthroplasty (RTSA) is often used to treat massive rotator cuff tears and nonspecific arthritis in people aged 65 and older [1]. In younger age groups, usually it is required only when there are comminuted fractures and arthroscopic repair of the periarticular structures has failed [2,3,4]. RTSA is performed in all of these situations to relieve pain and restore joint functionality [5,6]. Given the expansion of life expectancy and the ever-expanding proportion of the population experiencing shoulder injuries, it is estimated that RTSA surgery will increase by 92% by 2025 [5]. Already in the USA, more than 22,000 operations are performed annually [7,8]. Machine learning (ML) and computational intelligence (CI) methods, as well as other complex predictive algorithms, are now widely used so that they can provide all that information to achieve the best results of orthopedic surgeries like RTSA [9,10]. Several attempts have been made to integrate artificial intelligence (AI) in the software of robotic surgery devices to decrease the future failures and to increase the efficiency of shoulder surgical operations [11,12,13,14]. In the case of the RTSA, previous work has been performed in this field by grouping all the data from the pre- and post-surgery imaging controls to reduce the possibility of human error [15,16,17] such as hematomas, fractures of the humerus, as well as appropriate design in the angle and slopes of the implant [18]. However, while there are several studies on the creation of models that serve placement, size, assessment of postoperative range of motion, patient satisfaction, and recurrences, the field of classification and prognosis on rehabilitation after surgery is poor [17].

Usually, ML and CI methods divide a given dataset into two subsets, the training set and the test set, which are used for the training and the testing of the corresponding algorithms, respectively. Although, in recent years, there has been an ever-increasing trend of applying ML and CI methods in various scientific fields and especially in medicine, as far as we know there is no

- accurate classification algorithm;
- prognosis algorithm;
- development and application of ML and CI algorithms

for postoperative patients in the rehabilitation phase. To our knowledge, no similar research has been conducted in previous years. The objective of this study is to develop and apply ML and CI algorithms that, with the use of the minimum training set, can achieve the maximum performance in terms of classification accuracy and prognosis on the test set.

## 2. Materials and Methods

### 2.1. Data Description

The study was retrospective, and patient consent did not apply to this research design. For the purpose of the research, data were collected on features as gender, age, type of surgery, passive range of available motion (ROM) of all movements (preoperative and postoperative), visual analog pain scale (preoperative and postoperative), number of physical therapy sessions, and session duration. The data were collected at the Arthroscopy and Shoulder Surgery Center, which operates within the 3rd Orthopedic Clinic of the Hygeia Group, Athens, Greece. Measurements in all parameters were performed in the week before surgery, in the first trimester, as well as six months after surgery. In total, 120 patients, from 2015 to present, who underwent RTSA were included. Conversely, patients with revisions of previous shoulder surgery, shoulder girdle and cervicothoracic spine pathologies, hemiarthroplasty, humerus fracture, cancer, concomitant neurological diseases, intellectual disability, or dementia were excluded. These data were gathered in strict compliance with the provisions on General Data Protection Regulation, they were anonymized, and the anonymized data consisted of the material for the application of ML and CI algorithms. The study was approved by the Research Ethics and Deontology Committee of the Democritus University of Thrace, Thrace, Greece (58134/541/12-7-2023).

For each patient (case), the values of 17 features were recorded. These 17 features are codified and explained in Table 1.

Passive range of available motion of all movements of shoulder were recorded using a digital goniometer [19].

The total recovery time was used as a clustering attribute. Therefore, in total, the dataset comprised 120 cases (patients) × 17 attributes (features). Due to the small number of cases, binary transformation was used on the 17th attribute (total recovery time). If the value of the 17th attribute was less than 4.5 months, the corresponding instance was clustered into cluster 0. If the value was equal to or greater than 4.5 months, then the instance was clustered into cluster 1. This way, a binary classification problem emerged. Six different binary classification algorithms were developed and applied on these data. These algorithms were divided into two application phases, namely Phase I and Phase II, respectively, that are presented in the following.

### 2.2. Phase I Machine Learning Classifiers

The ML methods that were developed and applied on the RTSA dataset were K-nearest neighbors’ algorithm (KNN) and K-means clustering algorithm. The dataset was divided into two subsets, the training set and the test set. As a first step, both algorithms were trained with the use of the training set in order to construct the corresponding trained classification models. Afterwards, as a second step, the trained models were tested with the use of the test set. By these means, the application of these two ML methods concerned the investigation of the dependence of classification performance on the size of the training set.

KNN is a supervised nonparametric method, where no assumptions are made about the nature of the data. The algorithm utilizes a distance metric, such as the Euclidean distance, the Manhattan distance, or the Mahalanobis distance, in order to locate the *K* nearest training points that correspond to the cases of the training set in the feature space [20]. The points that are in closest proximity to a new case are referred to as neighbors. The classification of a new case is based on the corresponding classification of the majority of the *K* nearest neighbors. The one and only free parameter of the algorithm is *K*, i.e., the number of the nearest neighbors that are considered, in order to classify a new case [21]. In the computer experiments of the present work, we used various values of *K* (3, 5, 7, 11, 13) and the Euclidean distance.

On the other hand, the K-means clustering algorithm iteratively divides the points in the feature space, which correspond to the cases of the training set, into *K* predefined, distinct, and non-overlapping clusters. Each point is assigned to a unique cluster, based on its proximity to the clusters’ centroids. These centroids are recalculated at each iterative step, and the algorithm is expected to converge to the centroids and the corresponding clusters that minimize the intra-cluster variance [22]. The one and only free parameter of the K-means clustering algorithm is the number *K* of the clusters, which is predefined by the user. In the computer experiments reported in the present work, *K* was set to 2, given that the algorithm was applied on a binary classification task.

In both cases, the algorithms used the data of the training set in order to classify the data of the test set, and the algorithms’ classification performance was evaluated on the data of the test set. This classification of the test set cases was the output of both algorithms. These outputs were compared to the desired output, that is, to what classification the algorithm should give for the cases of the test set. Given the classification of each case of the test set, there are four possible outcomes, namely *true positive* (*tp*), *true negative* (*tn*), *false positive* (*fp*), and *false negative* (*fn*). The performance metric adopted in the present study was the *classification accuracy*, calculated as

*accuracy* = *(tp* + *tn)/(tp* + *tn* + *fp* + *fn).* (1)

### 2.3. Phase I Genetic Algorithm Clustering

In addition, GAClust, a clustering algorithm based on genetic algorithm (GA) search and optimization [23], was developed and employed. GA has been proven as a powerful tool in search and optimization, especially in cases where the search space is huge [24]. In the present study, the GAClust algorithm used the training set data and evolved the two clusters and the coordinates of the corresponding clusters’ centroids in the feature space in order to form two clusters in such a way that the classification error of the data of the test set, with respect to these centroids, was minimized. Consequently, the classification performance of GAClust on the test set was estimated by the use of (Equation (1)). Considering that the training set was of size *S*, i.e., it consisted of *S* cases, the binary genome of the GA was of length *L* = *S*. Each gene (corresponding to a binary digit) could be either 0 or 1, indicating the cluster that the corresponding case of the training set is classified. Using this binary codification for the GA genome, the GA population can be evolved to converge to the genome that codifies the grouping of the cases of the training set into two clusters. After calculating the coordinates of these two clusters’ centroids in the feature space, the algorithm classifies the cases of the test set by estimating the proximity of the cases to the two centroids. The outcome of this grouping is compared to the desired classification of the cases of the test set and the classification performance is calculated with the use of (Equation (1)). Therefore, as fitness function *f*_1_ of GAClust algorithm, the following calculation was used:

*f*_1_ = *acc*, (2)

where *acc* is the classification *accuracy* on the test set, according to (Equation (1)). For the evolution of the GA population, roulette-wheel selection, uniform crossover operator, and simple binary mutation operator were used for the employment of the selection, the crossover, and the mutation procedure, respectively. In all computer experiments, the size to the GA population ranged from 20 to 50 individuals, the crossover probability was set to 0.8, and the mutation probability was set to 0.01. *Elitism* was also activated, that is, the two fittest individuals of each generation were reproduced as clones to the next generation.

### 2.4. Phase II—Minimizing the Size of the Training Set

In Phase II, the research question was modified and the investigation focused on the optimization of the training set both in terms of its size (the smaller, the better) and its classification performance (the higher, the better). Therefore, three (3) hybrid algorithms were developed, namely GAKNN, GAK-means, and GA2Clust. These three hybrid algorithms were based on the three simple algorithms of KNN, K-means, and GAClust of Phase I and utilized GA search in order to find the smallest possible training set; with the use of the training procedure of KNN, k-means, and GAClust, it achieved the maximum classification performance on the test set. Considering that *N* = 120 is the total number of cases, and *S* is the number of cases in the training set, then there are

(3) $$\left(\begin{array}{c} N\\ S \end{array}\right)=\frac{N!}{\left(N-S\right)!S!}$$

distinct combinations to select *S* cases out of *N*. For example, given that in most of the ML applications, usually the 70% of the available data are used in the training set and the remaining 30% of the data in the test set, for *N* = 120, which corresponds to the total number of cases in the present study, by setting *S* = 0.7 *N* = 84, (Equation (3)) results to approximately 5^30^ combinations. Given that, in general, the size *S* of the training set could range from 1 to *N −* 1, the total number of potential combinations is

(4) $$\sum_{S=1}^{N-1}\left(\begin{array}{c} N\\ S \end{array}\right).$$

By combining (Equation (3)) to (Equation (4)), it is obvious that the size of the search space for the optimal training set is huge, therefore the implementation of efficient search and optimization methods, such as GA, is essential. Using binary codification for the GA genome with length *L* = *N*, each gene with value 1 codifies that the corresponding case is included in the training set and it is not included in the test set; otherwise, a gene value 0 codifies that the corresponding case is not included in the training set and it is included in the test set. This codification results in a search space of 2*^L^* = 2*^N^* = 2^120^ *≈* 10^36^ possible combinations. Given that the size of the training set is *S* and the GA has two objectives, to search (i) for the minimum training set and (ii) for the maximum classification performance, the fitness function *f*_2_ for the hybrid algorithm was defined as

(5) $$f_{2}=acc-alpha\frac{S}{N}$$

where *acc* is the classification accuracy according to (Equation (1)), *S* is the size of the training set, *N* is the total number of cases in the dataset, and *alpha* is a weight factor that balances *S* with respect to *acc*. Interpreting (Equation (5)), the higher the classification performance and the lower the size *S* of the training set, the larger the fitness function *f*_2_. In all computer experiments, the GA evolved a population of 100 individuals; roulette-wheel selection, uniform crossover, and binary mutation were used as selection, crossover, and mutation operators, respectively. The crossover probability was set to 0.8, the mutation probability was set to 0.01, and *elitism* was also activated for the two fittest individuals of each generation.

## 3. Results

### 3.1. KNN Algorithm

**Figure 1:**
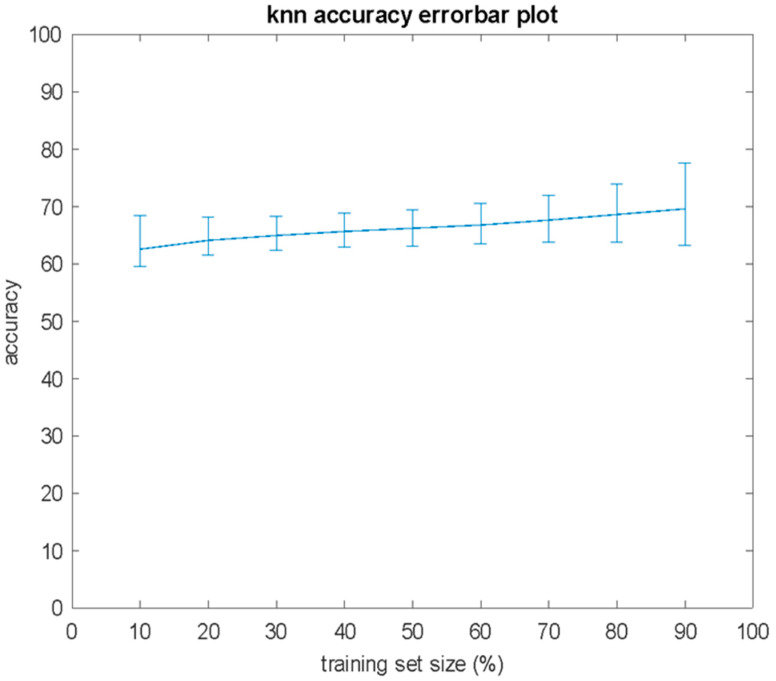
Mean value and standard deviation of classification accuracy versus the percentage of the training set size for 100,000 independent computer experiments of KNN algorithm, and *K* = 3.

**Figure 2:**
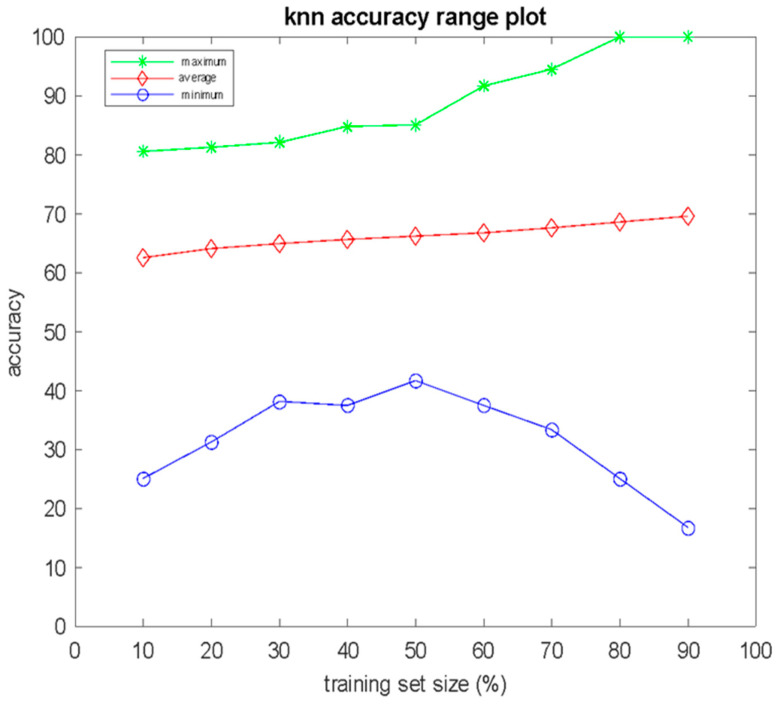
Mean value, maximum value, and minimum value of classification accuracy versus the percentage of the training set size for 100,000 independent computer experiments of KNN, and *K* = 3.

Numerous independent computer experiments were conducted for various values of the free parameter *K* of the KNN algorithm. In total, 100,000 independent experiments were conducted, and the mean value, the standard deviation, the minimum vale, and the maximum value of the classification performance, in terms of accuracy, according to (Equation (1)), were estimated for the size of the training set *S* in the range from 10% to 90%, with an increase step 10%. The obtained results for *K* = 3 are presented in Figure 1 and Figure 2. In Figure 1, in the form of an error bar plot, the averaged classification performance and standard deviation over 100,000 independent computer experiments are shown, in which v is the size *S* of the training set. As can be observed in Figure 1, the mean value of classification accuracy shows a slight increasing trend as the size of the training set increases. In Figure 2, there the average (red), maximum (green), and minimum (blue) classification performance of the 100,000 independent computer experiments are shown, with respect to the size *S* of the training set. As shown in Figure 2, there training sets with size 80% or 90% of *N* were found, which achieved 100% classification accuracy on the test set. To be more specific, for *S* = 0.8 *N*, four combinations of the training set that achieved accuracy = 100% were found, whereas the corresponding result for *S* = 0.9 *N* was that 921 combinations achieved accuracy = 100%. An additional point that can be seen in Figure 2 is that the deviation between the maximum and minimum values of the classification accuracy widened significantly as the size of the training set increased.

### 3.2. K-Means Algorithm

**Figure 3:**
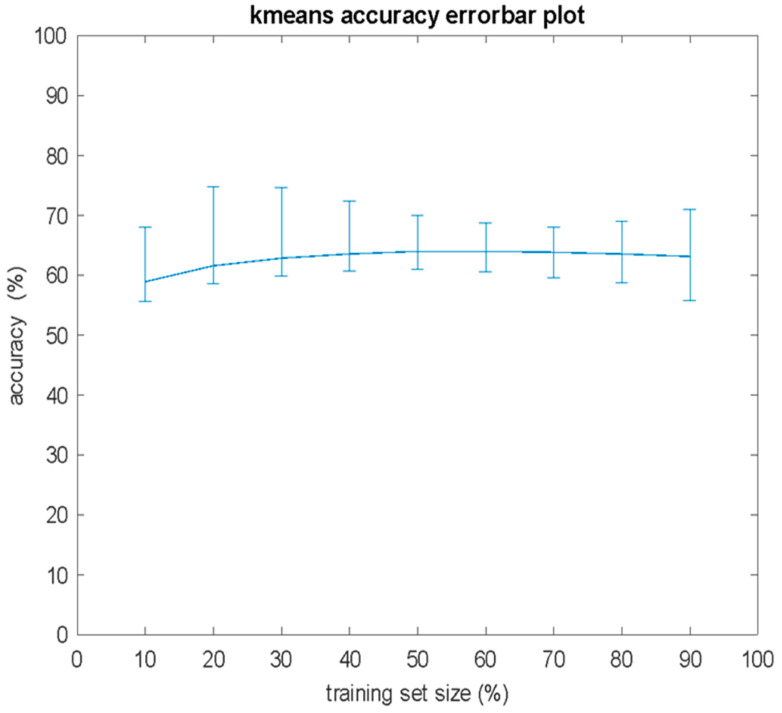
Mean value and standard deviation of classification accuracy versus the percentage of the training set size for 100,000 independent computer experiments of K-means algorithm.

**Figure 4:**
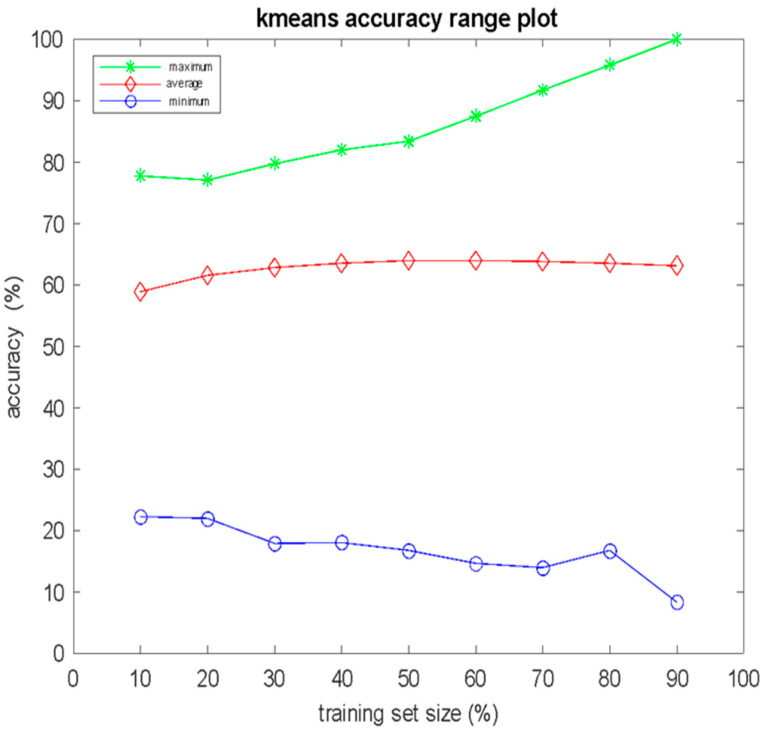
Mean value, maximum value, and minimum value of classification accuracy versus the percentage of the training set size for 100,000 independent computer experiments of K-means algorithm.

As in the case of the KNN, 100,000 independent experiments were conducted for the K-means algorithm, with training set sizes ranging from 10% to 90% of the total cases *N*, with a step size of 10% increase. With respect to the size *S* of the training set, in Figure 3, the the error bar plots of the mean value and the standard deviation are presented, whereas in Figure 4, the mean, maximum, and minimum values of performance on the test set are presented. The K-means algorithm achieved 100% accuracy in 265 experiments, all for *S* = 0.9 *N*. By combining the findings presented in Figure 3 and Figure 4, it can be seen that despite the fact that the mean performance remained almost unaffected by the size *S* of the training set, the deviations between the minimum and maximum values of the classification performance increased as *S* increased.

### 3.3. GAClust Algorithm

**Figure 5:**
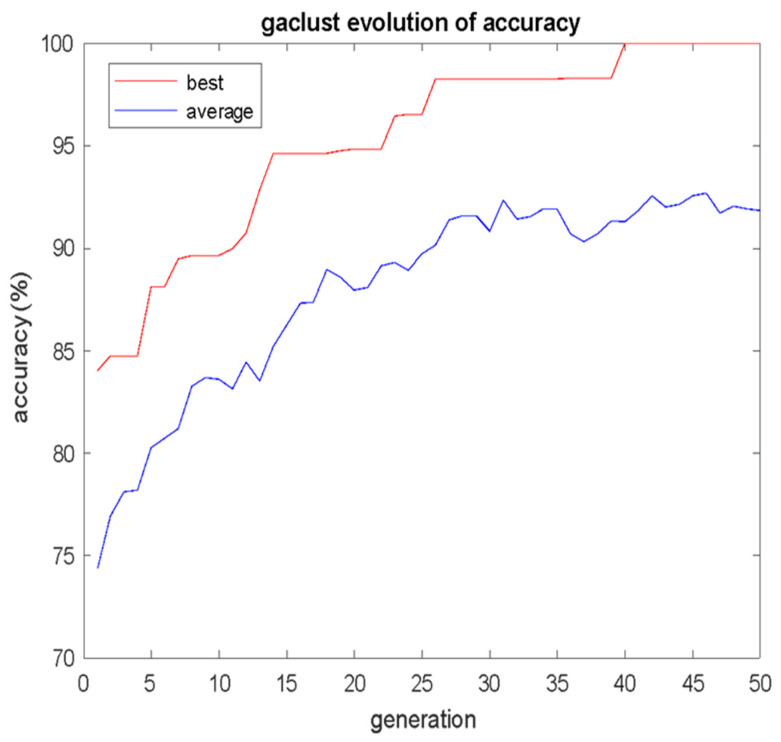
Evolution of mean and best fitness (accuracy) per GA generation.

Indicative results for a GA population of just 20 individuals that had evolved for 50 generations are as follows. The evolution of the mean fitness of each generation and the corresponding evolution of the best fitness of each generation are shown in Figure 5, where it is shown that the GAClust algorithm very rapidly converged to 100% accuracy. For the fittest individual of the last (50th) generation, the training set was constructed of *S* = 64 cases, as shown in Table 2. The 64 cases of Table 2 correspond to 53.33% of the total size of the dataset, and it is considerably smaller than 70% that is usually used ML training procedure.

### 3.4. GAKNN 

**Figure 6:**
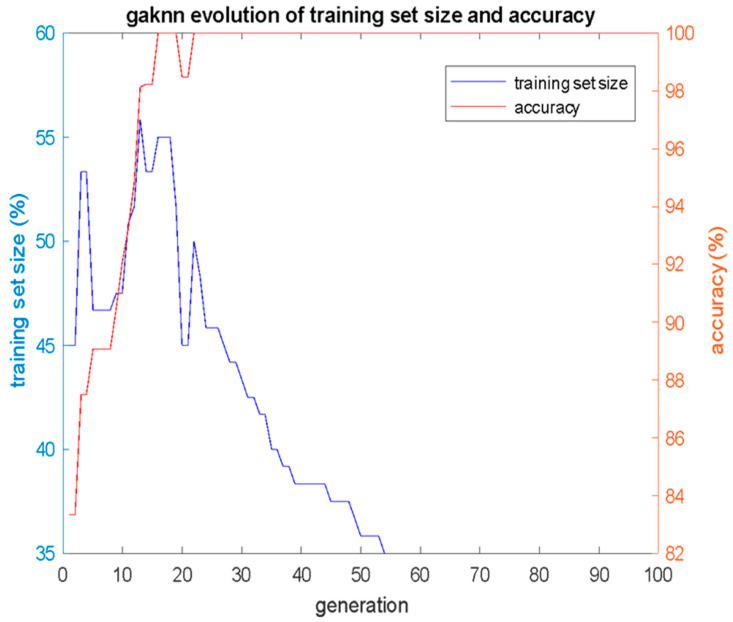
Evolution of classification accuracy *acc* and training set size *S* per GA generation for GAKNN, with *K* = 3 and *alpha* = 0.28.

In computer experiments of the GAKNN, the GA population of 100 individuals had evolved for 100 generations. *K* was set equal to 3 and the *alpha* weight factor in (Equation (5)) was set to 0.28. Given that the GA is a two-objective one searching simultaneously for the smallest size of the training set and the maximum classification performance, according to (Equation (5)), in Figure 6, the evolution of the training set size *S* of the fittest individual of each generation (blue line with respect to the blue right y-axis of the plot) are shown, as well as the corresponding classification accuracy *acc* of that individual (red line with respect to the red left y-axis of the plot). Figure 6 clearly illustrates the simultaneous increase in classification accuracy and the decrease in size *S* of the training set as the GA population evolves. The GAKNN converged to a training set that was consisted of 42 cases (42/120 = 35% of the total available data); therefore, the test set was consisted of the rest 78 cases (78/120 = 65%). The 42 cases that constructed the optimal training set for the GAKNN are shown in Table 3.

### 3.5. GAKmeans Algorithm

**Figure 7:**
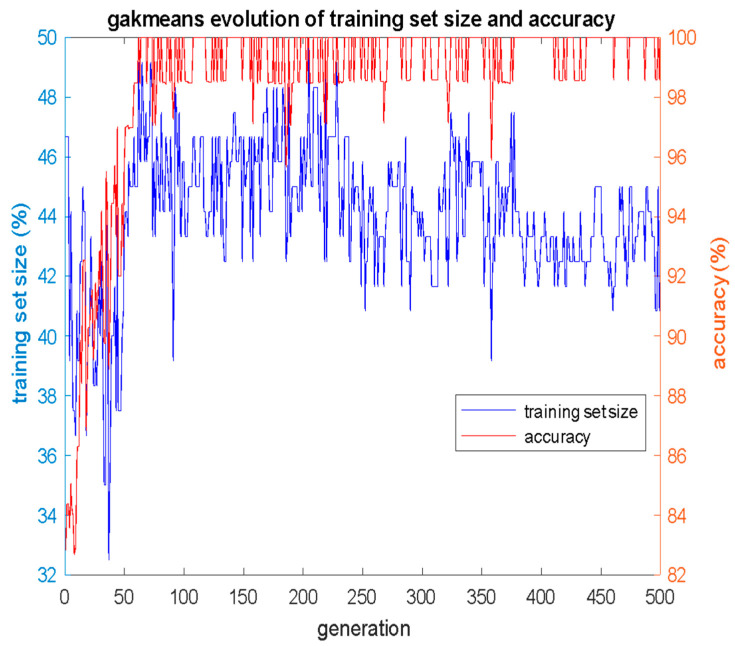
Evolution of classification accuracy *acc* and training set size *S* per GA generation for GAK-means algorithm, with *alpha* = 0.50.

The corresponding results obtained by the GAKmeans algorithm are presented in the same manner, in Figure 7 and Table 4. The *alpha* weight factor in Equation (5) was set to 0.5. The GAKmeans evolved a population of 100 individuals for 500 generations and converged to classification accuracy 100% with the use of just 50 cases in the training set, that is, *S* corresponds to 50/120 = 41.67% of the total size of the available data, which is considerably smaller than 70% that is usually used in ML experiments. The 50 cases that constructed the optimal training set for the GAKmeans algorithm are shown in Table 4.

### 3.6. GA2Clust Algorithm

**Figure 8:**
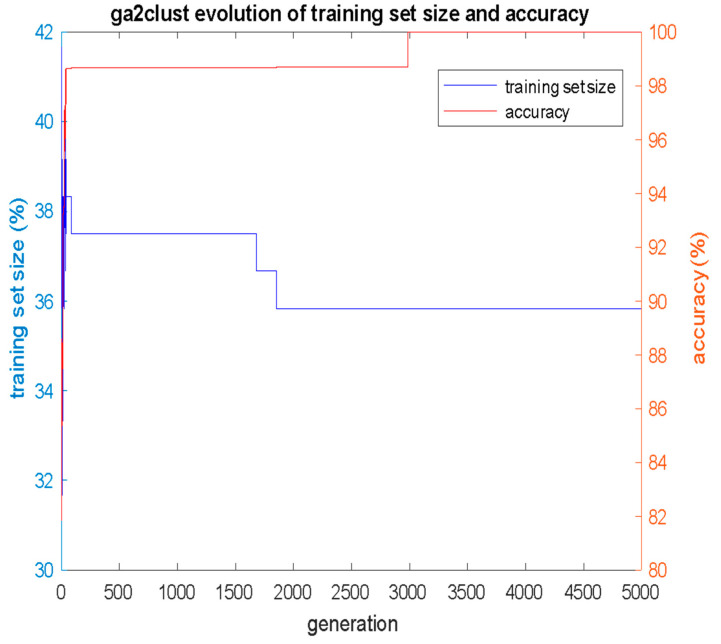
Evolution of classification accuracy *acc* and training set size *S* per GA generation for GA2Clust algorithm, with *alpha* = 0.625.

The results for the GA2Clust algorithm were obtained with a GA population of 500 individuals and 5000 generations of evolution. The *alpha* weight factor in (Equation (5)) was 0.625. As shown in Figure 8, 100% accuracy was achieved using only *S* = 43 cases in the training set (43/120 = 35.83%), while the remaining 77 incidents (77/120 = 64.17%) were used in the test set. Therefore, while the simple GAClust algorithm used 64 cases (53.33%), the GA2Clust algorithm used just 35.83% of the cases, meaning that the GA-based search reduced the required size S of the training set by 53.33% − 35.83% = 17.5% over the entire dataset. The optimal training set that the GA2Clust converged to is shown in Table 5.

## 4. Discussion

Unlike other studies that provided surgeons with feedback to improve their technique, our study assumed that the surgery was performed optimally for all patients and focused on rehabilitation. The different approaches between surgeons, mainly concerning the surgical techniques and methods, can affect the postoperative rehabilitation of the patients [25].

To maintain consistency and avoid variability in outcomes, we employed the surgical technique of a single surgeon. This approach minimizes the potential bias that could arise from different doctors reporting on their patients’ conditions [26].

The collected data were from 120 cases, each of which contained all relevant features for our examination. Despite identifying numerous features, only 17 were utilized to prioritize essential elements while minimizing the data required for learning. Although this number may appear small, comparable successful studies have used even smaller samples [27], validating Kymar’s conclusion [26] that both comprehensive and abbreviated machine learning models demonstrate similar accuracy in predicting clinical outcomes following RTSA at various postoperative intervals.

All measurements were obtained with scapula stabilization, passive motion by the examining physician, and using a digital goniometer [19].

In the literature, there are many different protocols that the physical therapist can follow in the post-operative rehabilitation of patients who have undergone RTSA [7]. Essentially, in reverse total shoulder arthroplasty, the center of rotation of the joint is shifted medially and downward, increasing the lever arm of the deltoid [28]. It is therefore needed to activate the deltoid muscle and to strengthen it afterwards [4,29]. Since most of these patients are elderly, the immobilization period may adversely affect the outcome of the operation [4]. Early mobilization is intended to help reduce stiffness and establish a rapid functional outcome [4]. The risk of instability is high [1,4]. The rates of instability are reported in the first trimester postoperatively due to early mobilization involving 1/3 of patients who usually require surgical revision [7,30].

This period can vary from the 2nd to the 6th postoperative week and is combined, depending on the case, with breathing exercises, shoulder girdle exercises, and trunk stabilization [7,31]. In this phase of maximal protection, the patient can passively or assistedly move the upper extremity up to 90 degrees of flexion and perform pendulum movements [28]. Usually around the 12th week after surgery, active movement follows and continues with strengthening [28]. In most patients, the phase of rehabilitation takes about 138 days, which corresponds to about 4.5 months [28]. We also used this threshold for the binary transformation of our instances.

The protocol followed by the patients included, for the first 3 weeks, mobilization of peripheral shoulder joints such as fingers, wrist, and elbow, as well as movements of the shoulder girdle. Afterwards, for a period ranging from 3 to 9 weeks, which depended on pain and patient compliance, patients performed assisted, hanging, and pendulum exercises. Subsequently, once they had achieved most of the range of motion in all the main movements, for the next 3 weeks, the exercise was active and combined with proprioceptive exercises. We then suggested strengthening exercises until full recovery was attained. The total rehabilitation time ranged from 4 to 6 months. It is worth noting that patients, on a case-by-case basis, had to add breathing exercises, trunk–scapula stabilization, and/or stretching to the rehabilitation program.

The patients who took part in the research had at least one weekly meeting with the physical therapist and then repeated these exercises at home throughout the week. Many researchers argue that instructing patients to perform the exercises continuously at home is more effective in increasing ROM than having them perform the exercises in the physical therapy clinic with supervision [4]. These data have been recorded for the participants in the study and used subsequently.

There have been previous attempts by other colleagues to use an algorithm with a wearable device or through a smartphone application to measure ROM and detect non-positive rehabilitation progress early [15,32,33].

There were two main objectives of the present study. First, to develop and apply ML algorithms on RTSA data, a task that has not been covered in the literature so far. For that task, two well-known and widely used algorithms were utilized, namely the KNN and the K-means algorithm. Both these two algorithms managed to achieve 100% classification performance using, respectively, 80% and 90% of the available data for training. For the purpose of comparison, a third classification algorithm was developed, namely the GAClust algorithm that performed GA clustering. The obtained results indicated that GAClust overperformed K-means as well as KNN, because GAClust utilized only 53.3% of the available data in the training set in order to achieve 100% classification performance. From these findings emerged the second task of the present work, related to the search for the minimum training set that can achieve 100% classification performance. For this second task, three hybrid CI algorithms were developed, namely GAKNN, GAKmeans, and GA2Clust, where GA search was utilized in order to optimize the training set and the classification performance simultaneously. All these three hybrid algorithms overperformed the simple ML algorithms and the simple GAClust algorithm by providing results indicating that 100% classification performance can be achieved even with use of 35% of the entire dataset.

## 5. Conclusions

The proposed hybrid computational intelligence algorithms outperformed simple machine learning algorithms for RTSA classification and rehabilitation prognosis in terms of optimization of the size of essential data needed for their training procedure as well as the reduction in the computational cost.

Specifically, the simple ML algorithms in Phase I achieved 100% classification and prediction performance on the test set, using 90% of the available training data. The hybrid CI algorithms based on genetic algorithm optimization in Phase II achieved 100% performance on the test set, using only 35.83% of the available training data.

They can also be used as a computational intelligence tool for evaluating patients postoperatively during rehabilitation by completing scores in each direction of motion as well as the measuring time, and they detect non-positive rehabilitation progress early.

## 6. Limitations

There were no scores for all examinees, such as ASES or CONSTANT SCORE assessment, so these data were also not included. Also, the cases of patients who stopped rehabilitation due to complications or did not complete 6 months were also not included.

## Figures and Tables

**Table 1:** RTSA rehabilitation recorded features.

Nr.	Code	Feature
**1**	**Age**	Age at the time of surgery (years)
**2**	**FF pre**	Forward Flexion pre-operatively
**3**	**HyprE pre**	Hyperextension pre-operatively
**4**	**Ab pre**	Abduction pre-operatively
**5**	**Ex r pre**	External rotation pre-operatively
**6**	**In r pre**	Internal rotation pre-operatively
**7**	**FF post3**	Forward Flexion 3 months post-operatively
**8**	**HyprE post3**	Hyperextension 3 months post-operatively
**9**	**Ab post3**	Abduction 3 months post-operatively
**10**	**Ex r post3**	External rotation 3 months post-operatively
**11**	**In r post3**	Internal rotation 3 months post-operatively
**12**	**FF post6**	Forward Flexion 6 months post-operatively
**13**	**HyprE post6**	Hyperextension 6 months post-operatively
**14**	**Ab post6**	Abduction 6 months post-operatively
**15**	**Ex r post6**	External rotation 6 months post-operatively
**16**	**In r post6**	Internal rotation 6 months post-operatively
**17**	**Trt**	Total rehabilitation time (months)

**Table 2:** Cases in training set used by GAClust algorithm.

1 4 5 7 8 10 11 12 13 14 15 17 18 20 21 24 25 26 29 30 31 33 34 36 38 43 45 49 51 54 55 58 59 61 62 66 67 70 72 73 74 76 77 78 80 82 84 85 86 88 89 92 93 95 96 97 98 101 102 103 109 115 116 118

**Table 3:** Cases in training set used by GAKNN.

1 3 6 9 11 21 26 29 31 33 41 43 47 49 51 52 53 54 56 61 62 63 66 67 71 73 78 81 82 83 84 86 89 90 93 97 99 102 103 108 115 120

**Table 4:** Cases in training set used by GAKmeans algorithm.

1 2 5 6 7 8 11 14 15 17 18 21 23 28 29 31 32 34 38 41 43 45 57 59 62 66 67 69 70 72 73 74 77 78 79 80 81 82 84 92 97 98 101 102 103 104 110 112 115 117

**Table 5:** Cases in training set used by GA2Clust algorithm.

1 4 6 10 11 15 17 19 21 29 30 31 33 34 42 43 45 51 54 57 62 65 66 70 73 74 77 78 80 82 84 85 89 92 96 97 98 102 103 105 109 112 115

## Data Availability

Data are available upon request, by contacting by e-mail the correspondence author.
